# Supplementary material for: Towards sustainable land management in small islands: A Water-Energy-Food nexus approach
Source: PLoS One. 2024 Nov 19;19(11):e0310632. doi: 10.1371/journal.pone.0310632 (PMC11575787; doi:10.1371/journal.pone.0310632)
Supplement: Attachment — Submitted filename: Response to Reviewers (1).docx [file pone.0310632.s001.docx]

**Response to Reviewers**

Dear editor,

I would like to express my gratitude to you and the reviewers for providing valuable feedback on our manuscript titled *“Towards sustainable food systems in small islands: a
Water-Energy-Food nexus approach”.* We appreciate the time and effort invested in evaluating our work.

In response to the reviewers' comments, we have carefully addressed each point raised and made the necessary revisions to improve the quality of the manuscript. Below, we provide a point-by-point response to the reviewers' comments:

Elements added to the manuscript	Elements already present in the manuscript

**MAIN COMMENTS OF REVIEWER 1**

***There is no clear explanation about the optimization model. There is no mention of it in the text, only in figure 2, which says it is coded in python. According to table 1, the optimization model seems to be one of partial equilibrium, but it is no clear at all.***

The model is more detailed in a previous paper (refer to **lines 231-232** in the manuscript).

Please refer to **Table 2** in the **Appendix** section for the corresponding equation specifications of the constraints mentioned.

We have added clarifications at **lines 298-299** in the manuscript.

***There are no comments about model "validation" or the likelihood of model outputs. In my opinion it is a good practice that a ms like this include a section in which the authors explain why the model is reliable, and why we should be confident with its results.***

Elements on the use of intervals to manage uncertainties related to data are found at **lines 306-308**

We have added clarifications at **lines 309-318.**

***There is no discussion about the "economic aspects" of changing crops, and this is a very important issue in sustainability topics. In this context, perhaps it would be interesting to include a section that explores the "transformation effort" to increase the SSR.***

We have chosen to exclude economic aspects from our approach and we explain why at **lines 663-669.**

***I think that some comments are needed about the relevance of this system considering the worldwide sustainability issue. Planet Earth is a very small island too, so conclusion of this research may be very interesting for addressing sustainability issues in a larger scales.***

To justify that our conclusions are applicable on a larger scale, we draw an analogy with the donut theory at **lines 625-640.**

***It would be interesting to include a small comparisons of the WEF approach with other efforts, like FABLE. Why WEF is a good choice for studying sustainability issues ?***

The relevance of a WEF nexus approach is justified at **lines 676-680.**

**PARTICULAR COMMENTS OF REVIEWER 1**

***Perhaps modify figure 3, in order to show where in Earth is Reunion Island***

For the sake of clarity of **figure 3**, which we feel is important, we prefer not to add a new figure on top of or next to it. Instead, we detail where Reunion is located at **lines 352-354.**

***I could not find the footnotes of figures!***

The footnote has been integrated into the text at **lines 491-492.**

***I could not find figure 10***

Included in the PDF just after **Fig9.**

***The reference #35 seems to be incomplete. This is a very important reference for the*** ***ms***

Modifications have been made at **lines 798-801.**

***Perhaps join figures 5 to 8 into a single one, that would make easier the comparison of*** ***scenarios***

According to us, joining figure 5 to 8 into a single one would not facilitate the interpretation of the graph. We consider that having a graph for each scenario gives a better idea of the threshold effects for each scenario and each case (best case, worst case).

***Perhaps include a short name in the S1, S2, S3 and S4, so the text would be easier to read. For example: "S1 (traditional BAU, business as usual)"***

Names have been added to the scenarios for greater clarity. **Fig4** has been modified and we find the inclusion of abbreviated names for scenarios in figure legends between **line 558 and line 559**. Inclusion in the text at **lines 584, 602, 603, 608, 609.**

**Perhaps include a graph like this:
+ in X axis the time (yrs);
+ in Y axis the SSR value;
+ a point showing the actual SSR value of Reunion island;
+ time trajectories of each scenario, showing their final SSR values.
This will be more easy to understand**

Thresholds are studied for long-term projections 🡪 2035. The production of curves with time on the x-axis and SSR on the y-axis has already been done in the previous paper (please refer to **reference 35 at lines 798-801**).

***Include an appendix with a more detailed explanation of interviews (how many, main results of them, que questions posed, etc.)***

The main questions asked during the interviews have been added at **lines 435-436 / lines 447-452.**

***Figure 9 is too simple, data do not deserve a graph***. ***Perhaps if the 80% portion is separated in individual crops would be worthwhile to keep***

For information only. It shows the proportion of the population with a traditional or Mediterranean diet, which is an indicator of the potential for transition from one diet to another. In our opinion, the diagram is important to keep in order to catch the reader's eye.

**MAIN COMMENTS OF REVIEWER 2**

***Authors should discuss the results and how they can be interpreted from the perspective of previous studies and working hypotheses. The results and their implications should be discussed in the broadest possible context.***

To justify that our conclusions are applicable on a larger scale, we draw an analogy with the donut theory at **lines 625-640.**

***Future research directions may also be highlighted.***

Please refer to **lines 681-685** for future research directions.

***The authors should briefly mention the limitation of their study.***

The main limitations of the study are mentioned at **lines 663-676.**

**JOURNAL REQUIREMENTS**

1) Our manuscript appears to meet PLOS ONE’s style requirements.

2) The funding information has been removed from the **Acknowledgements** section at **lines 687-688.**

4) **Fig 3** was produced by the author. We have added clarifications at **lines 360-363.**

Additionally, we have revised and corrected the English of the article. Thank you once again for your consideration of our manuscript. We are committed to making any further revisions deemed necessary to enhance the quality and clarity of the manuscript.

Sincerely,

**AUTHIER Romain**
