# Supplementary material for: Towards sustainable land management in small islands: A Water-Energy-Food nexus approach
Source: PLoS One. 2024 Nov 19;19(11):e0310632. doi: 10.1371/journal.pone.0310632 (PMC11575787; doi:10.1371/journal.pone.0310632)
Supplement: Attachment — Submitted filename: Response_to_reviewers.docx [file pone.0310632.s002.docx]

**Response to Reviewers**

Dear editor,

I would like to express my gratitude to you and the reviewers for providing valuable feedback on our manuscript*.* We appreciate the time and effort invested in evaluating our work.

**Response to reviewer 1:**

All comments have been addressed.

**Response to reviewer 2:**

Following the concerns of the second reviewer, we revised the title of our paper, to match more closely our contributions focused on the land management issues, and avoid potential confusions or misinterpretations with more food or agriculture production-based research. The new title is: *Towards sustainable land management in small islands: a Water-Energy-Food nexus approach.*

Thank you for your consideration of our manuscript. We are committed to making any further revisions deemed necessary to enhance the quality and clarity of the manuscript.

Sincerely,

AUTHIER Romain
